# Postoperative Complications After Acute Achilles Tendon Rupture Repair: A Survival Analysis of Minimally Invasive vs Open Techniques

**DOI:** 10.1177/10711007251333777

**Published:** 2025-04-28

**Authors:** Joris R. H. Hendriks, Siddhartha Sharma, Matthias Peiffer, Tom M. de Groot, Gregory Waryasz, Gino M. M. J. Kerkhoffs, Soheil Ashkani-Esfahani, Christopher W. DiGiovanni, Daniel Guss

**Affiliations:** 1 Foot & Ankle Research and Innovation Lab (FARIL), Department of Orthopaedic Surgery, Massachusetts General Hospital, Harvard Medical School, Boston, MA, USA; 2 Department of Orthopedic Surgery and Sports Medicine, Amsterdam UMC, University of Amsterdam, Amsterdam, the Netherlands; 3 Department of Orthopedic Surgery, University Medical Center Groningen, Groningen, the Netherlands; 4 Foot and Ankle Center, Department of Orthopaedic Surgery, Massachusetts General Hospital, Newton-Wellesley Hospital, Boston, MA, USA; 5 Amsterdam Movement Sciences, Sports, Musculoskeletal Health, Amsterdam, the Netherlands; 6 Academic Center for Evidence-based Sports Medicine (ACES), Amsterdam, the Netherlands; 7 Amsterdam Collaboration on Health & Safety in Sports (ACHSS), IOC Research Center, Amsterdam, the Netherlands

**Keywords:** Achilles rupture complications, Achilles rupture, MIS, survival analysis

## Abstract

**Background:**

The complication rates after surgical repair of acute Achilles tendon ruptures (ATRs) using open repair or minimally invasive surgical techniques (MIS) have been debated extensively. Despite significant research, a literature hiatus exists on the timing of these complications between techniques. In this study, we aimed to address this gap by conducting a Kaplan-Meier survival analysis to compare the incidence and timing of complications after open vs MIS repair of acute ATRs and examine associated risk factors.

**Methods:**

This retrospective study included patients ≥18 years who underwent surgical treatment of an acute ATR within 28 days of injury and had a minimum of 90-day follow-up. Demographics, surgical technique (open vs MIS repair), and the occurrence and timing of postoperative complications were collected. Postoperative complications were classified as venous thromboembolism, rerupture, surgical site infection, wound dehiscence, and sural nerve injury. A Kaplan-Meier curve was employed to compare the complication rates between groups. The log-rank test was used to test the equality of survivor functions. The Cox proportional hazards model was used to determine predictors of complications.

**Results:**

In total, out of 417 patients, 52 complications were reported in 50 patients. We found no significant difference in the complication rates between the MIS and open repair groups. Cox proportional hazards modeling revealed that BMI was a significant predictor of rerupture (HR 1.2, 95% CI 1.05-1.4) and that surgical delay increased the risk of wound dehiscence (HR 1.2, 95% CI 1.01-1.3) and sural nerve injury (HR 1.2, 95% CI 1.1-1.3).

**Conclusion:**

MIS and open repair techniques for acute ATRs demonstrate comparable complication rates. However, patients with elevated BMI exhibit a modest increased risk of rerupture, regardless of the technique used. Those with surgical delay beyond 2 weeks are also modestly more likely to experience wound dehiscence with open surgical approach and sural nerve injury among MIS-treated patients.

**Level of Evidence:** Level III, retrospective cohort study.

## Introduction

Acute Achilles tendon ruptures (ATRs) can result in significant loss of function.^10,15^ Although the relative benefit of surgical treatment as compared to nonoperative management continues to be debated, there has been heightened interest in the use of minimally invasive techniques (MIS) to mitigate rates of wound complications.^2,29,31^ The incidence of wound dehiscence and surgical site infections (SSIs), as well as other complications such as sural nerve injury and tendon rerupture after open and minimally invasive surgery (MIS) techniques, are variable in the literature.^4,6,21,22,29^ Indeed, a recent meta-analysis of randomized controlled trials comparing open and MIS repair of acute ATRs found several methodological issues, including small sample size, low study power, large variability in the follow-up duration, and not considering confounding factors.^4^

The Kaplan-Meir analysis, also known as “survival analysis,” has been widely used in cancer studies to study survival rates. This technique determines not only the occurrence of events but also the estimated time of the event. Furthermore, it allows for analyzing confounding factors that could potentially affect the event rate.

This study aims to perform a survival analysis comparing the rates and timing of complications after open vs MIS techniques for acute ATR. Additionally, we investigated the influence of several important confounding variables that could potentially affect the complication rates.

## Materials and Methods

### Study Design and Participants

After the institutional review board approval (IRB no. 2015P000464), we retrospectively gathered data on patients who underwent surgical treatment of an acute ATR between 2015 and 2021 at 3 tertiary referral hospitals in Boston, MA. The current study was conducted in accordance with the Strengthening the Reporting of Observational Studies in Epidemiology (STROBE) guidelines.^12^ Patients were included if they were (1) 18 years or older, (2) had undergone surgical treatment for an acute ATR within 28 days of injury, and (3) had a follow-up duration of at least 90 days. Patients were excluded if they had a documented history of Achilles tendinopathy, ruptures beyond 28 days, those with reruptures, and patients with other traumatic injuries to the foot and ankle. A formal a priori power analysis was not conducted, as this was a retrospective cohort study using all available eligible patients. However, a post hoc analysis was performed to determine whether the sample size was adequately powered to determine differences between the 2 groups.

### Variables and Outcome Measures

Patient baseline demographics, including sex, age, body mass index (BMI), and smoking status (current/former/never), were collected, as were clinical variables, including the type of surgical treatment (MIS or open repair), laterality, time to surgery (in days), and follow-up duration (in days). Smoking status was categorized as smokers (current) and nonsmokers (former, never). The time to surgery was determined by subtracting the injury date from the surgery date, as recorded in the examination notes. Comorbidities were assessed using the Charlson Comorbidity Index.^9^ Medication details were also evaluated, with special reference to fluoroquinolones, glucocorticoids, and statins, as these drugs have been shown to increase the risk of tendon ruptures.^14,32,34^

Outcome variables included rerupture, wound dehiscence, surgical site infections (SSIs), sural nerve injury, and venous thromboembolism (VTE). The time to each complication was also recorded. If a patient had more than 1 complication, each complication was factored in as an individual event. Rerupture was defined as a postoperative break in the continuity of the tendon, confirmed either by ultrasonographic or MRI examination. Wound dehiscence was defined as a breach in the skin extending up to the dermis or deeper. SSIs were defined in accordance with the Musculoskeletal Infection Society (MSIS) criteria, and were classified as either deep (necessitating wound debridement) or superficial (oral or intravenous antibiotic therapy only).^5,27^ Sural nerve injury was defined as numbness, reduced sensation, or tingling in the sural nerve dermatome. VTE was classified as a deep venous thrombosis diagnosed on color Doppler examination or a pulmonary embolism on CT pulmonary angiography.

### Surgical Technique

The open repair technique consisted of an approximately 8-10-cm posteromedial incision overlying the Achilles with the identification of the paratenon as a separate layer for subsequent closure. Tendon ends were minimally debrided, and tendon ends were opposed using a running, locking, nonabsorbable suture.

The MIS repair technique consisted of a 2-cm longitudinal incision overlying the rupture site with the paratenon identified for subsequent closure.^35^ Intrasheath adhesions were gently released with a Cobb or malleable retractor. Thereafter, a nonproprietary, ringed clamp was passed in a subparatenon fashion, and straight Keith needles were used to sequentially pass a nonabsorbable suture percutaneously through the ringed clamp and tendon ends and then pulled into the wound, rendering the sutures subparatenon. The tendon ends were tied with the ankle held in equinus.

Each patient received a standardized rehabilitation protocol, with follow-up visits scheduled at 2 weeks, 2 months, and 6 months. The postoperative protocol was identical for both the MIS and open surgery approaches. Complications were evaluated during these routine follow-up visits, emergency department visits, or through patient-initiated calls if they noticed any postoperative concerns.

### Statistical Analysis

The Kaplan-Meier method was used to determine the median survival time and 95% CIs for each outcome variable of interest. Survival curves were plotted, and the log-rank test was used to compare the equality of survivor functions between the MIS and open repair groups. Outcomes are presented as mean ± SD/SE or median (interquartile range) where applicable. *P* values of <.05 were considered as significant for the log-rank test. A Cox proportional hazards model was employed to account for confounding factors, and hazard ratios with CI were determined for each variable of interest. The proportionality assumption was checked before performing the Cox modeling; this was done by plotting the log(–log(survival)) against the survival time for different levels of covariates. All analysis was conducted via Stata MP, 17.0.

## Results

### Baseline Characteristics

**Figure 1.**
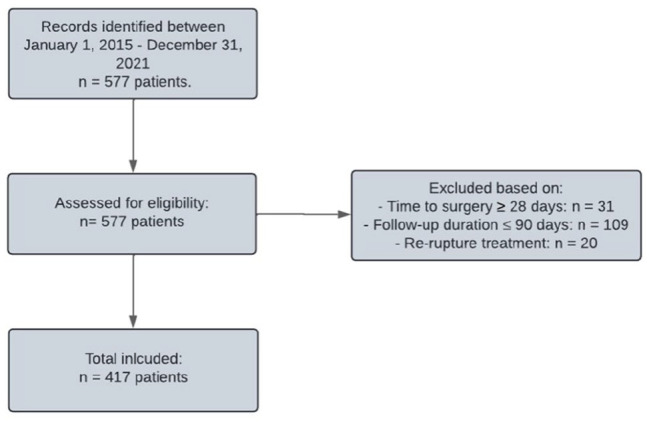
Flowchart illustrating the inclusion of the study population.

A total of 417 patients were included (Figure 1). Among these patients, the median age was 39 years (IQR, 31-48), 342 patients (82.0%) were male, and 267 patients (64.1%) were treated with MIS. The MIS group was noted to have a significantly shorter follow-up duration than the open repair group (*P* = .04; Table 1). There was no significant difference in other baseline characteristics.

**Table 1.** Baseline Characteristics of the Study Cohort.

Variable	MIS (n = 267)	Open (n = 150)	*P* Value^a^
Age, y, mean ± SD	40.3 ± 12.6	40.7 ± 11.9	.80
BMI, mean ± SD	26.9 ± 4.1	27.1 ± 3.8	0.76
Time to surgery, d, mean ± SD	9.4 ± 5.2	9.4 ± 5.9	.70
Gender			.06
Male	226	116	
Female	41	34	
Smoker			.37
Yes (current smokers)	17	6	
No (former, or never smokers)	250	144	
Length of follow-up, d, mean ± SD	228.4 ± 163.6	284.2 ± 309.7	**.04**

Abbreviations: BMI, body mass index; MIS, minimally invasive surgery.

a Boldface indicates significance (*P* < .05).

### Complication Rates

During the follow-up period, 52 complications in 50 patients (12.5%) were recorded (Table 2). The most common complication was venous thromboembolism (5.3%), followed by sural nerve injury (2.6%), surgical site infection (2.4%), reruptures (1.2%), and wound dehiscence (1.0%). The overall incidence rate per person-day was 0.02 for all complications, 0.0002 for VTE, 0.0001 for sural nerve injury, 0.0001 for surgical site infection, 0.00005 for reruptures, and 0.00004 for wound dehiscence. Of the 10 patients who sustained an SSI, 2 had superficial infections that needed oral antibiotic therapy and 8 had deep infections that needed irrigation, debridement, and intravenous antibiotics.

**Table 2.** Comparison of Complication Rates Between MIS and Open Surgical Repair.

Complication	Total Events, n	Overall Incidence Rate, %	MIS Group (n = 267)		Open Group (n = 150)		*P* Value^a^
			Total Events, n	Incidence Rate, %	Total Events, n	Incidence Rate, %	
Venous thromboembolism	22	5.3	17	6.4	5	3.3	.18
Reruptures	5	1.2	3	1.1	2	1.3	.86
Wound dehiscence	4	1.0	2	0.75	2	1.3	.58
Surgical site infection	10	2.4	4	1.5	6	4	.12
Sural nerve injury	11	2.6	10	3.8	1	0.7	.06
All complications^b^	52	12.5	36	13.5	16	10.7	.35

Abbreviation: MIS, minimally invasive surgery.

a *P* value was obtained from the log rank test.

b One patient had both infection and sural nerve injury, and hence this was counted as a single event for determining all complications.

Cox proportional hazards modeling (Table 3) did not reveal a significant difference in the overall complication risk between MIS and open repair (HR 0.73, 95% CI 0.34-1.53, *P* = .41). Similarly, gender, age, BMI, smoking status, time to surgery, length of follow-up, Charlson Comorbidity Index, and VTE prophylaxis were not noted to be significant predictors of overall complications.

**Table 3.** Results from Cox Proportional Hazards Modeling.

Variable	Hazard Ratio	SE	*Z*	*P* Value^a^	95% CI
All complications					
MIS vs open	0.73	0.28	−0.83	.41	0.34-1.53
Gender	0.70	0.26	−0.96	.34	0.34-1.45
Age	1.00	0.02	0.33	.74	0.97-1.03
BMI	0.97	0.03	−1.08	.28	0.92-1.02
Smoker	0.72	0.38	−0.63	.53	0.26-1.99
Time to surgery	1.00	0.03	0.21	.83	0.96-1.06
Length of follow-up	1.00	0.001	−0.79	.43	0.99-1.00
CCI	0.92	0.24	−0.32	.75	0.54-1.54
Drug intake	1.00	-	-	-	-
VTE prophylaxis	0.39	0.32	−1.16	.25	0.08-1.91
Venous Thromboembolism					
MIS vs open	0.51	0.27	−1.28	.20	0.18-1.42
Gender	0.84	0.48	−0.31	.76	0.28-2.55
Age	1.05	0.03	1.76	.08	0.99-1.10
BMI	1.04	0.05	0.71	.48	0.94-1.42
Smoker	2.56	1.98	1.22	.22	0.56-11.64
Time to surgery	0.93	0.04	−1.50	.13	0.85-1.02
Length of follow-up	1.00	0.00	0.85	.40	1.00-1.002
CCI	0.90	0.27	−0.35	.73	0.50-1.62
Drug intake	0.00	0.00	0.00	>.99	0.00
VTE prophylaxis	1.39	1.44	0.32	.75	0.18-10.67
Reruptures					
MIS vs Open	1.37	1.39	0.32	.75	0.19-9.91
Gender	1.35	1.58	0.25	.80	0.13-13.51
Age	0.94	0.06	−0.86	.39	0.83-10.7
BMI	1.22	0.09	2.66	**.01**	1.05-1.42
Smoker	0.00	–	–	–	–
Time to surgery	0.78	0.11	−1.72	.09	0.58-1.04
Length of follow-up	1.00	0.00	−0.01	.99	1.00-1.003
CCI	0.00	0.00	0.00	>.99	0.00
Drug intake	0.00	–	–	–	–
Wound dehiscence					
MIS vs Open	1.30	1.37	0.25	.80	0.16-10.31
Gender	0.75	0.90	−0.24	.81	0.07-7.81
Age	1.04	0.07	0.54	.59	0.90-1.19
BMI	1.07	0.12	0.63	.53	0.86-1.43
Smoker	0.00	0.00	0.00	>.99	0.00
Time to surgery	1.15	0.08	1.99	**.047**	1.001-1.31
Length of follow-up	1.00	0.00	0.12	.91	1.00-1.004
CCI	0.87	0.74	−0.17	.87	0.16-4.59
Drug intake	0.00	0.00	0.00	>.99	0.00
Surgical site infection					
MIS vs open	2.40	1.63	1.29	.20	0.63-9.11
Gender	1.34	1.13	0.34	.73	0.26-7.00
Age	0.97	0.04	−0.76	.45	0.90-1.05
BMI	0.92	0.09	−0.81	.42	0.76-1.12
Smoker	0.00	0.00	0.00	>.99	0.00
Time to surgery	0.90	0.08	−1.24	.22	0.76-1.06
Length of follow-up	1.00	0.00	1.64	.10	1.00-1.002
CCI	1.29	0.70	0.47	.64	0.44-3.76
Drug intake	0.00	0.00	0.00	>.99	0.00
Sural nerve injury					
MIS vs Open	0.16	0.17	−1.69	.09	0.02-1.32
Gender	0.36	0.25	−1.47	.14	0.09-1.41
Age	1.02	0.04	0.47	.64	0.95-1.09
BMI	1.07	0.06	1.13	.26	0.95-1.20
Smoker	3.58	2.72	1.68	.09	0.81-15.89
Time to surgery	1.11	0.05	2.44	**.02**	1.02-1.21
Length of Follow-up	1.00	0.00	−0.17	.86	1.00-1.003
CCI	1.11	0.40	0.29	.77	0.55-2.26
Drug intake	0.00	0.00	0.00	>.99	0.00

Abbreviations: BMI, body mass index; CCI, Charlson Comorbidity Index; MIS, minimally invasive surgery; VTE, venous thromboembolism.

a Significant values are highlighted in bold.

For tendon reruptures, BMI was a significant predictor (HR 1.22, 95% CI 1.05-1.42, *P* = .01), indicating that each unit increase in BMI was associated with a 22% higher risk of rerupture. No other variables, including time to surgery and surgical approach, were significantly associated with rerupture risk. The mean BMI was 31.6 ± 11.14 in patients who experienced a rerupture, compared with 27.0 ± 3.99 in those without rerupture.

For wound dehiscence, time to surgery was a significant predictor (HR 1.15, 95% CI 1.001-1.31, *P* = .047), suggesting that each additional day of surgical delay increased the risk by 15%. No other variables, including BMI and surgical approach, were significantly associated with wound dehiscence. The mean time to surgery was 9.32 ± 4.93 days in patients with wound dehiscence, compared with 9.41 ± 5.47 days in those without.

For sural nerve injury, time to surgery was a significant predictor (HR 1.11, 95% CI 1.02-1.21, *P* = .02), indicating that each additional day of delay increased the risk by 11%. No other variables were significantly associated with sural nerve injury. The mean time to surgery was 10.39 ± 5.77 days in patients with sural nerve injuries, compared with 9.42 ± 5.47 days in those without.

For venous thromboembolism and surgical site infections, none of the variables, including surgical approach, gender, BMI, time to surgery, or smoking status, reached statistical significance. (Appendices 1-6).

### Post Hoc Power Analysis

Post hoc power analysis demonstrated that our study had 83.5% power to detect a moderate effect size (Cohen *d* = 0.3) for differences in complication rates between MIS and Open repair groups. This suggests that our sample size was adequate for detecting clinically meaningful differences.

## Discussion

The debate continues about the relative benefit of MIS vs open repair as the preferred surgical ATR repair techniques. Although some studies have reported higher complication rates with one approach over the other, findings have been inconsistent, further obscuring the current consensus on the optimal technique. Moreover, although the incidence of complications has been the focus in most previous studies, the time-to-event has yet to be explored. In our study, we have therefore performed a survival analysis comparing open repair and MIS, which considers confounding factors.

To our knowledge, this study has one of the highest number of patients (ie, 417) available in the current literature, substantially adding to the growing body of evidence of MIS vs open repair. Previous retrospective studies ranged from 19 to 615 patients.^8,11,13,16,20,25,33^

Our results indicate that there was no statistically significant difference between the 2 surgical techniques in terms of time-to-event and incidence rate for sural nerve injury, VTE, SSI, wound dehiscence, and reruptures. This finding is consistent with a previous RCT that also reported no significant difference in complication rates between both techniques.^28^ With respect to sural nerve injuries, previous research has shown that MIS is associated with an increased risk.^19,23^ In contrast, several previous studies have shown no significant difference in sural nerve injury rates for both approaches.^1,17^

With regard to VTE occurrence, this is not included in most previous studies. A previous RCT has reported no significant difference between the 2 techniques, with an occurrence of 1.1% and 0.6% for open repair and MIS, respectively.^28^ Our nonsignificant findings correspond to the current literature; however, higher VTE rates were found in our study compared with the literature (3.3% and 6.4%, respectively). Concerning rerupture rates, some studies have found a slightly increased risk for MIS techniques^24,26^; however, none of those presented significant results.^16^ This is in line with the nonsignificant result of our study. The mean overall rate of reruptures in previous studies for MIS and open repair was 2.4% and 2.1%, respectively.^16^ Results from our study (1.1% and 1.3%, respectively) show a comparable occurrence, although with a slightly higher risk in open repair. Regarding SSIs, most previous studies have shown an increased odds risk of 3.90 to 5.40 for open surgery compared with MIS.^4,16,17^ Our study could not confirm these findings, as no difference was found between both techniques. In a previous meta-analysis, the infection (deep and superficial combined) rate was 1.2% and 3.8% for MIS and open repair, respectively.^16^ Our results align with these findings, with an SSI rate of 1.5% and 4.0%, respectively. Although other studies have found obesity (BMI >30) to have an increased association with wound dehiscence and VTE after repair, it has not yet been investigated for rerupture risk.^7,18^ In the shoulder, elevated BMI has been shown to correlate with higher rerupture rates after rotator cuff repair.^3^ The authors hypothesized that this could be due to a higher mechanical strain on the repaired tendon.

Although the relationship between time-to-surgery and postoperative complications in Achilles surgery has not yet been defined, it has been established in ankle fracture surgery.^30^ Surgical delay leads to scarring and adherence not only of the tendon ends but also of the paratenon, as well as thickening and edema of the subcutaneous layers, making this harder to achieve. Future studies should investigate the influence of time to surgery in relation with postoperative complications.

Several limitations to our study should be noted. Although the average follow-up was 7.5 months in the MIS group and 9.5 months in the open group, the minimum follow-up duration was 90 days. Many complications, such as wound healing issues or VTE, are likely captured in this time frame, but others, such as rerupture, may extend beyond this. We did not observe the patients long enough to determine whether sural nerve injury was permanent or temporary. Also, we could not differentiate between symptomatic vs nonsymptomatic VTE in our study based on the clinical notes because it was not clear to us whether the suspicion of the clinician was due to pain, swelling, and other signs and symptoms of VTE, or it was due to the history of the patient (previous reports of such complications) or clinical judgment. This might have caused a higher rate of VTE in our study compared with the reports in the literature. Because of a low population in our complicated cases, we could not come up with reliable cut-off values for the correlated factors, which suggests future studies with larger populations to overcome this limitation. Furthermore, the data were collected retrospectively through our registry and thus did not include randomization, which could induce selection bias. This selection bias is not only patient-specific but also provider-specific, given that the majority of orthopaedic foot and ankle surgeons within our system preferentially use MIS techniques, whereas surgeons of other specialties often gravitate toward an open technique. Prior studies have demonstrated that surgeon subspecialty is a predictor of wound complication rates, though in this study, we found no difference.^33^ Furthermore, we did not include patient-reported outcome measurements and functional scores or return to sport, which withholds us from making conclusions on the clinical superiority of one technique over the other. Our groups presented asymmetry, as the MIS group contained significantly more subjects than the open repair group. Furthermore, included patients underwent surgery by different surgeons from our institution that might affect the outcomes based on different surgeons’ variations in experience and expertise. Although this may contribute to a lack of consistency in the results, this increased the sample size of our study significantly. In addition, this also enhances our findings’ generalizability.

## Conclusion

The results of this study indicate that MIS and open repair techniques for acute ATRs demonstrate comparable complication rates. However, patients with elevated BMI exhibit an increased modestly increased risk of rerupture, regardless of the technique undertaken. Patients with surgical delay have a small elevated likelihood of wound dehiscence in an open surgical approach and sural nerve injury in MIS-treated patients.

## Supplemental Material

sj-docx-2-fai-10.1177_10711007251333777 – Supplemental material for Postoperative Complications After Acute Achilles Tendon Rupture Repair: A Survival Analysis of Minimally Invasive vs Open Techniques Supplemental material, sj-docx-2-fai-10.1177_10711007251333777 for Postoperative Complications After Acute Achilles Tendon Rupture Repair: A Survival Analysis of Minimally Invasive vs Open Techniques by Joris R. H. Hendriks, Siddhartha Sharma, Matthias Peiffer, Tom M. de Groot, Gregory Waryasz, Gino M. M. J. Kerkhoffs, Soheil Ashkani-Esfahani, Christopher W. DiGiovanni and Daniel Guss in Foot & Ankle International

sj-pdf-1-fai-10.1177_10711007251333777 – Supplemental material for Postoperative Complications After Acute Achilles Tendon Rupture Repair: A Survival Analysis of Minimally Invasive vs Open Techniques Supplemental material, sj-pdf-1-fai-10.1177_10711007251333777 for Postoperative Complications After Acute Achilles Tendon Rupture Repair: A Survival Analysis of Minimally Invasive vs Open Techniques by Joris R. H. Hendriks, Siddhartha Sharma, Matthias Peiffer, Tom M. de Groot, Gregory Waryasz, Gino M. M. J. Kerkhoffs, Soheil Ashkani-Esfahani, Christopher W. DiGiovanni and Daniel Guss in Foot & Ankle International
