# Supplementary material for: Postoperative Complications After Acute Achilles Tendon Rupture Repair: A Survival Analysis of Minimally Invasive vs Open Techniques
Source: Foot Ankle Int. 2025 Apr 28;46(7):707–14. doi: 10.1177/10711007251333777 (PMC12227820; doi:10.1177/10711007251333777)
Supplement: sj-docx-2-fai-10.1177_10711007251333777 – Supplemental material for Postoperative Complications After Acute Achilles Tendon Rupture Repair: A Survival Analysis of Minimally Invasive vs Open Techniques [file sj-docx-2-fai-10.1177_10711007251333777.docx]

**Appendix**


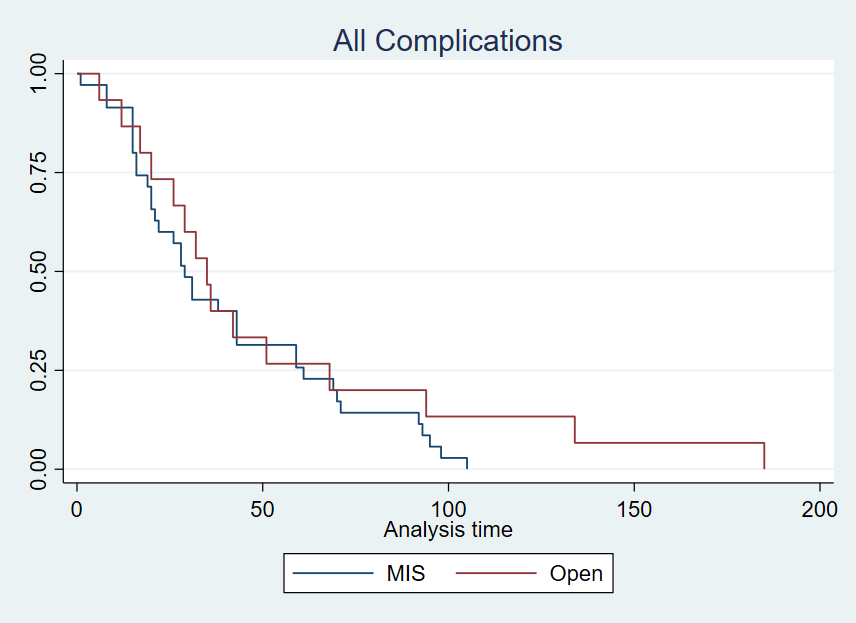


Appendix 1. This figure shows the Kaplan-Meier survival plots, comparing the overall complication rates between MIS and open surgical repair for acute Achilles tendon ruptures. On the x-axis the analysis time is shown in days.


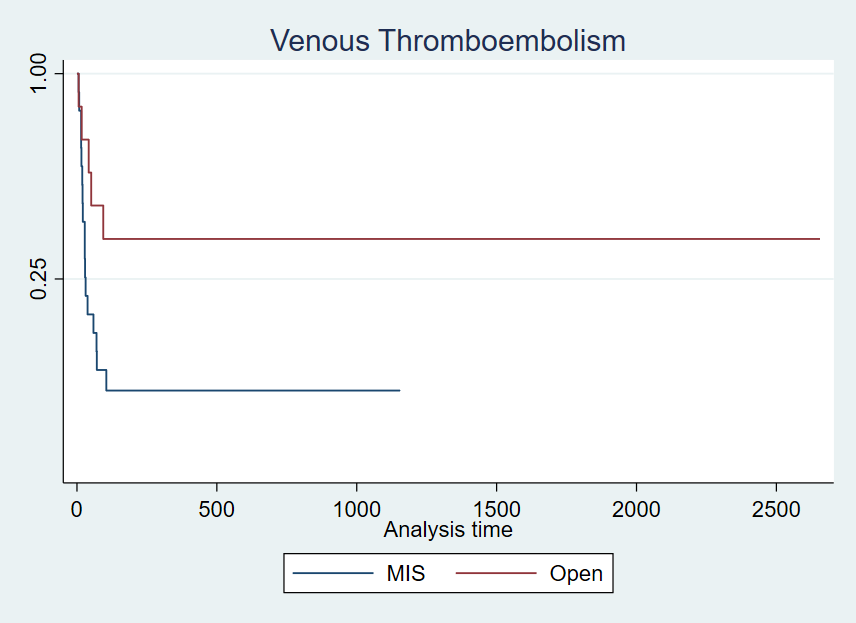


Appendix 2. This figure shows the Kaplan-Meier survival plots, comparing the venous thromboembolism rates between MIS and open surgical repair for acute Achilles tendon ruptures. On the x-axis the analysis time is shown in days.


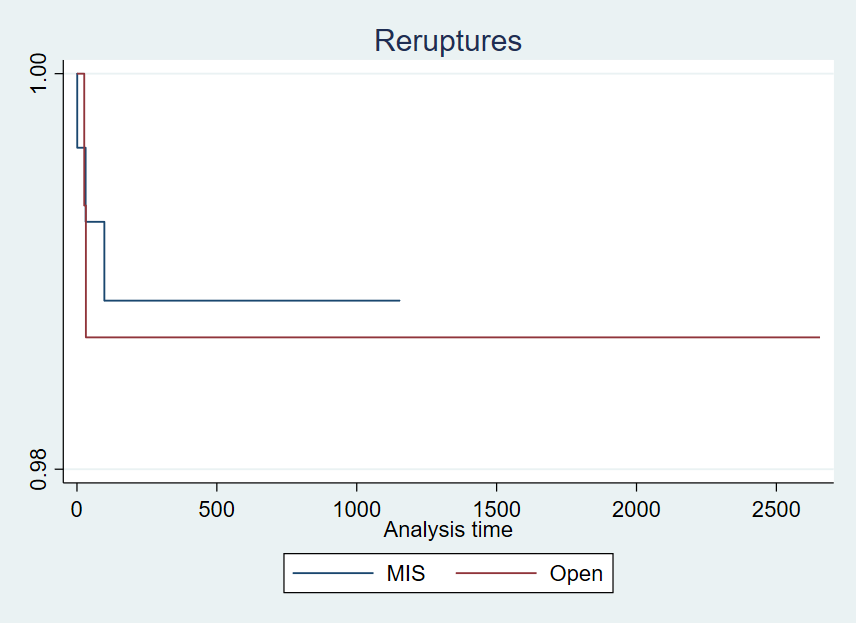


Appendix 3. This figure shows the Kaplan-Meier survival plots, comparing the re-rupture rates between MIS and open surgical repair for acute Achilles tendon ruptures. On the x-axis the analysis time is shown in days.


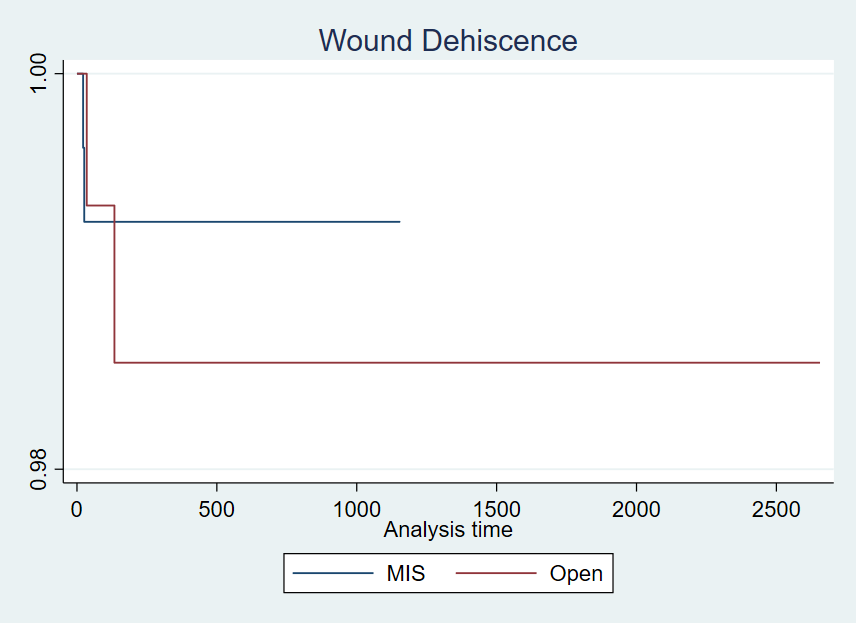


Appendix 4. This figure shows the Kaplan-Meier survival plots, comparing the wound dehiscence rates between MIS and open surgical repair for acute Achilles tendon ruptures. On the x-axis the analysis time is shown in days.


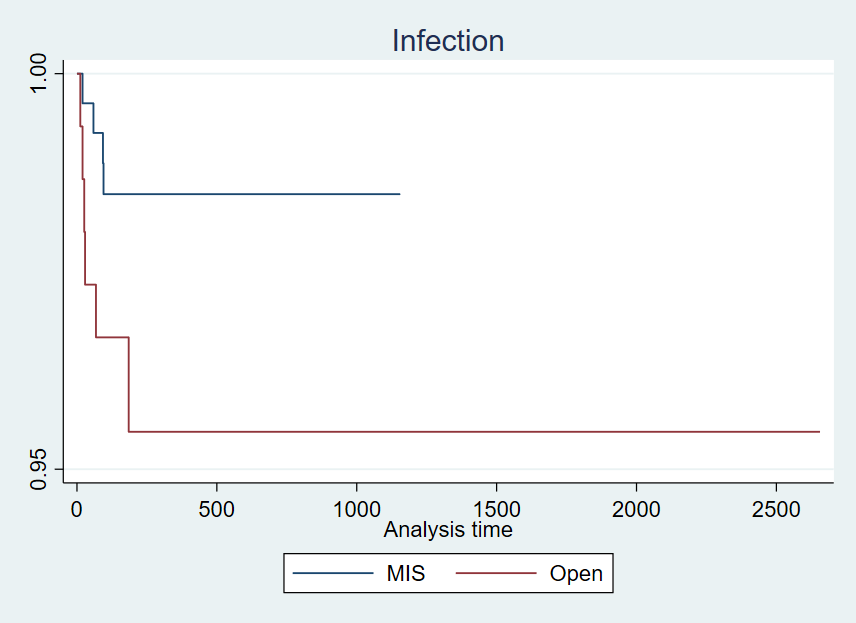


Appendix 5. This figure shows the Kaplan-Meier survival plots, comparing the surgical site infection rates between MIS and open surgical repair for acute Achilles tendon ruptures. On the x-axis the analysis time is shown in days.


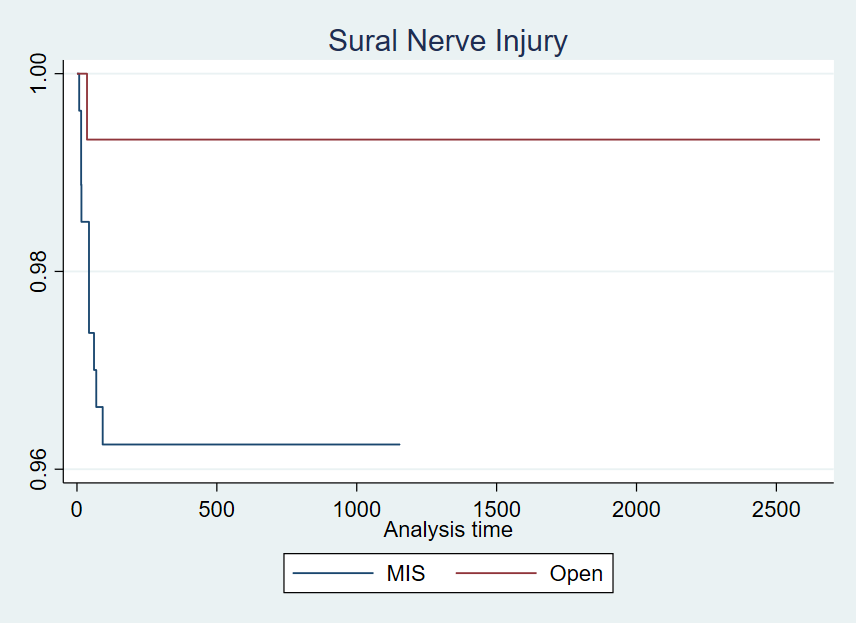


Appendix 6. This figure shows the Kaplan-Meier survival plots, comparing the sural nerve injury rates between MIS and open surgical repair for acute Achilles tendon ruptures. On the x-axis the analysis time is shown in days.
